# The Implications of Using a Physiologically Based Pharmacokinetic (PBPK) Model for Pesticide Risk Assessment

**DOI:** 10.1289/ehp.0901144

**Published:** 2009-09-24

**Authors:** Chensheng Lu, Christina M. Holbrook, Leo M. Andres

**Affiliations:** 1 Exposure, Epidemiology, and Risk Program, Department of Environmental Health, Harvard School of Public Health, Boston, Massachusetts, USA;; 2 Department of Environmental and Occupational Health, Rollins School of Public Health, Emory University, Atlanta, Georgia, USA

**Keywords:** chlorpyrifos, dietary pesticide exposure, PBPK, pesticide risk assessment, TCPY, urinary biomarker

## Abstract

**Background:**

A physiologically based pharmacokinetic (PBPK) model would make it possible to simulate the dynamics of chemical absorption, distribution, metabolism, and elimination (ADME) from different routes of exposures and, in theory, could be used to evaluate associations between exposures and biomarker measurements in blood or urine.

**Objective:**

We used a PBPK model to predict urinary excretion of 3,5,6-trichloro-2-pyridinol (TCPY), the specific metabolite of chlorpyrifos (CPF), in young children.

**Methods:**

We developed a child-specific PBPK model for CPF using PBPK models previously developed for rats and adult humans. Data used in the model simulation were collected from 13 children 3–6 years of age who participated in a cross-sectional pesticide exposure assessment study with repeated environmental and biological sampling.

**Results:**

The model-predicted urinary TCPY excretion estimates were consistent with measured levels for 2 children with two 24-hr duplicate food samples that contained 350 and 12 ng/g of CPF, respectively. However, we found that the majority of model outputs underpredicted the measured urinary TCPY excretion.

**Conclusions:**

We concluded that the potential measurement errors associated with the aggregate exposure measurements will probably limit the applicability of PBPK model estimates for interpreting urinary TCPY excretion and absorbed CPF dose from multiple sources of exposure. However, recent changes in organophosphorus (OP) use have shifted exposures from multipathways to dietary ingestion only. Thus, we concluded that the PBPK model is still a valuable tool for converting dietary pesticide exposures to absorbed dose estimates when the model input data are accurate estimates of dietary pesticide exposures.

The passage of the Food Quality Protection Act (FQPA; 1996) has streamlined research efforts devoted to assessing exposure to organophosphorus (OP) pesticides. As a result, our understanding of OP exposures and associated risk factors, particularly in children, has improved greatly (Fenske et al. 2002a; Koch et al. 2002; Lu et al. 2001, 2004, 2006, 2008; Whyatt and Barr 2001). However, our ability to use exposure measurements to estimate absorbed doses, which is essential for comparing exposures to toxicologic benchmarks such as the reference dose (RfD), remains limited.

A physiologically based pharmacokinetic (PBPK) model would permit simulation of the dynamics of chemical absorption, distribution, metabolism, and elimination (ADME) from different routes of exposures and, in theory, could be used as a tool for evaluating biomarker measurements, such as blood or urine levels, that are associated with the exposures (Rigas et al. 2001; Timchalk et al. 2002; Zhang et al. 2007). The mechanistic representation of biological processes embedded in PBPK models enables systematic route, dose, and species extrapolation. For these reasons, PBPK models have been applied in chemical risk assessments (Blancato 1994; Clewell et al. 2001) that are relevant for the interpretation of biomarker data.

The objective of this study was to use a PBPK model to validate measures of urinary excretion of 3,5,6-trichloro-2-pyridinol (TCPY), which is the specific metabolite for chlorpyrifos (CPF), in young children. Exposure and urinary biomarker data used in this study were obtained from a pesticide exposure assessment study that was designed specifically to assess daily aggregate exposure to OP pesticides in children. We evaluated the use of the PBPK model for applying aggregate exposure inputs and for estimating the cumulative urinary excretion with urinary biomarkers. The PBPK model outputs, in the form of predicted cumulative urinary excretion of TCPY in urine, were compared with the urinary TCPY levels measured in the four spot urine samples collected from each child. A consideration in the study design was that the results of the aggregate pesticide measurements would be of value for evaluating a PBPK-modeling approach by comparing the predicted and measured cumulative urinary metabolite excretion.

## Methods

### Child-specific PBPK model development

We initiated model development using PBPK models that were developed for CPF in rats and adult humans (Timchalk et al. 2002) in which CPF and chlorpyrifos oxon (CPFO) were modeled assuming circulation through physiologic well-mixed compartments. Metabolism of CPF (and CPFO) in the Timchalk et al. (2002) models was assigned to occur in the blood and liver, with the excreted metabolite, TCPY, lumped into a single compartment. To enable systematic extrapolation of the dose–urinary excretion behavior, we expanded the metabolite description from a volume-of-distribution concept to a physiologic description that can incorporate known differences at different life stages, such as between children and adults.

Physiologic parameters of the metabolic compartments and the partition coefficients were estimated by the same computational method (Poulin and Krishnan 1995). Glucuronic conjugation and the urinary excretion rate parameters were based on TCPY data in rats (Timchalk et al. 2005) and in adult humans (Nolan et al. 1984). The rat model was first adapted and compared with the data, and human parameters were applied when known. In cases where *a priori* values were not available, rat parameters were scaled by body weight (BW) to establish an initial value that was then adjusted based on model fit. The parameters relevant to the physiologic description of the metabolite are shown in Table 1. Differences in physiology between rats and adult humans are generally well known, whereas the kinetic constants were usually scaled by BW. The adaptations made to the adult PBPK model included modifications to the parameters for age, body volume, activity cardiac output, and alveolar ventilation rate. The cardiac output and alveolar ventilation rates were estimated using published equations (Price et al. 2003) and normalized to BW raised to the 3/4 power. The volumes for each individual tissue/organ were entered as a percentage of body volume, which was age adjusted. The parameters associated with the physiologic description of the metabolite in the child-specific PBPK model are shown in Table 2.

The rat, adult, and child PBPK models were implemented using the U.S. Enivironmental Protection Agency’s (U.S. EPA) Exposure Related Dose Estimating Model (ERDEM; Blancato et al. 2006). The ERDEM platform consists of two parts: the Microsoft Windows-based graphical user interface and the model engine, which is built in Advanced Continuous Simulation Language. The parameters for the study participants and for their exposure scenarios were created in ERDEM as the model data sets (MDSs) to ensure consistent implementation and to facilitate review. The differential equations associated with the PBPK model are described online [see Supplemental Material available online (doi:10.1289/ehp.0901144.S1 via http://dx.doi.org/). Zhang et al. (2007) successfully applied the ERDEM platform to develop the PBPK model for carbofuran, one of the cholinesterase-inhibiting carbamate insecticides.

### Human exposure data

The data used in the PBPK-model simulation were obtained from a cross-sectional pesticide exposure assessment study with repeated environmental and biologic sampling that was conducted in the U. S. state of Washington in 1998 (Fenske et al. 2002b; Kissel et al. 2005; Lu et al. 2004). Participants were recruited from a pool of children who had been enrolled in two previous assessment studies of pesticide exposure in Washington State (Koch et al. 2002; Lu et al. 2001).

**Figure 1:**
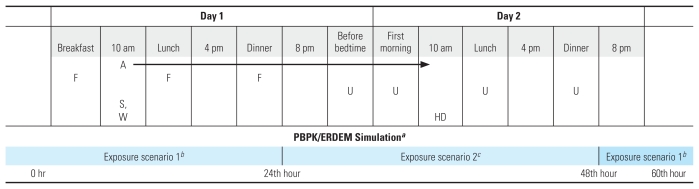
Sampling schedule for the 1998 study (1) and the corresponding PBPK model simulation (2). Abbreviations: A, 24-hr indoor air; F, 24-hr duplicate diets; HD, house dust; S, soil; W, hand/toy wipe; U, spot urine. The arrow indicates a 24-hr sampling. ***a***Modeled exposure duration for 168 hr; however, figure truncated after the 60th hr. ***b***Exposure scenario included rate ingestion and inhalation. ***c***Exposure scenario included rate ingestion, inhalation, and bolus food ingestion.

Children with elevated OP pesticide exposure based on the measurements of urinary dialkylphosphate levels from these two studies were targeted for the present study to increase the probability that participants would have detectable OP residues in their home or diet. We saw this strategy as a more efficient use of limited research resources than randomly selecting from hundreds of children whose OP pesticide exposure might fall below the analytical limits of detection. In brief, the study was conducted in the homes of 13 children 3–6 years of age who lived either in an urban or suburban area or in the agricultural region where OP pesticides were used in nearby fruit tree orchards. In 1998, each home was sampled twice, once in the summer (June–August) and again in the fall (October). Samples collected for exposure measurements included drinking water, outdoor soil, house dust, and toy wipe samples, in addition to indoor air and duplicate food samples over 24 hr. Four spot urine samples were collected over a 24-hr period (Figure 1). We selected CPF for modeling because it was commonly found in the environmental samples and its metabolite, TCPY, was frequently detected in the urine samples. The study protocol and procedures to obtain the assent of the children and informed consent of their parents or guardians were reviewed and approved by the University of Washington Institutional Review Board. We obtained informed consent from the study participants before initiating the study.

### Exposure scenarios

To account for the children’s aggregate exposure to CPF, each ERDEM/MDS was developed to allow data input for three concurrent exposure scenarios: bolus ingestion (based on levels measured in three meals throughout the sampling day), inhalation, and rate ingestion for nondietary routes (such as hand-to-mouth activity). The dermal route of exposure was not taken into account in the PBPK model simulation because CPF was not detected on any of the children’s hands at the time sampling was taking place.

#### Inhalation

Because 24-hr indoor air CPF concentrations were measured in a time-weighted average manner, we activated the static lung subsystem, instead of breathing lung, in each ERDEM/MDS. This activation required the input of the percent volume for the lung tissue (Fisher et al. 1998) and for the relevant partition coefficients. The blood/lung partition coefficient was assumed to be equal to that of the rapidly perfused tissue (4.33), and the blood/air partition coefficient was set as the large default value (10^9^) that represents nearly complete absorption (close to 100% because of the alveolar dead space) of inhaled OP pesticides and no exhalation of absorbed OP pesticides. These absorption characteristics are consistent with the low volatility of OP pesticides.

#### Rate ingestion

CPF exposure through the ingestion of contaminated soil and house dust was modeled as rate ingestion. The average daily rate of soil ingestion for children is 100 mg/day (U.S. EPA 2002a), and we assumed the same daily rate of house-dust ingestion for the PBPK/ERDEM simulations. Exposure through the ingestion of CPF residue transferred from toys to a child’s hands and to the mouth was also modeled as rate ingestion. We assumed that each child played (indoors and outdoors) for 2–2.8 hr each day (U.S. EPA 2002a) and that 7 hand-to-mouth transfers occurred in each hour of play that resulted in a conservative estimate of 20 hand-to-mouth activities while playing each day. We also assumed that 3% of the CPF residue measured on the child’s toy was transferred to the child’s hand after each contact (Clothier 2000) and estimated the total daily ingestion of CPF from toys via hand-to-mouth activity by multiplying the CPF residue measured on the toy surface by 0.6 (20 hand-to-mouth transfers × 3%).

#### Bolus ingestion

Duplicate food samples collected over 24 hr were combined to obtain a single meausre of dietary CPF intake over the sampling day. Although meals were knowingly consumed by the children at different time points throughout the day, dietary intake was modeled as a single bolus ingestion during dinner at 7 pm rather than continuously throughout the day. This decision was made because of the way the 24-hr duplicate food samples were composited to one large food sample for each child. Therefore, instead of arbitrarily spreading CPF residues measured in the 24-hr duplicate food samples across different eating events, we minimized the artificial variability and assigned it to a single bolus ingestion at 7 pm each day.

#### Biological monitoring

The timing of spot urine collection was tailored to capture OP pesticides through all possible exposure pathways (Figure 1). The first spot sample was collected before bedtime on the same day when the environmental and food sampling activities were completed (Day 1). Three additional spot urine samples were collected the next day (Day 2) during the first morning void, after lunch, and after dinner.

The PBPK model simulation period was determined based on the biological half-life of CPF, which is between 16 and 30 hr depending on the route of exposure (Griffin et al. 1999). Model simulations indicated that a half-life of 24 hr was appropriate for aggregate scenarios, and the total exposure duration was set for 168 hr, equivalent to 7 half-lives of CPF. We initiated the model simulation at hr 0 to account for the body burden of CPF resulting from previous exposures via inhalation or ingestion of house dust or soil (Figure 1). The exposure scenario for food ingestion was started at the 24th hr of the model simulation. The rate ingestion exposure duration was set to represent 12 hr of exposure (while awake) and 12 hr without exposure (while sleeping and inactive) every 24 hr throughout the entire exposure duration (168 hr).

#### PBPK model evaluation

The PBPK model outputs for each MDS included the predicted cumulative excretion of TCPY (mmol) in urine for the duration of exposure (168 hr), as well as the estimated absorbed dose for each MDS. The first 60-hr window of the model simulation was comparable to the actual urinary TCPY levels that were measured in the four spot urine samples and was the window of interest for model evaluation because of its accuracy. The measured cumulative mass of TCPY excreted in urine was calculated by multiplying the volume-weighted average of the four spot urine samples by 500 mL/day, the total daily urine excretion volume for children 2–5 years of age.


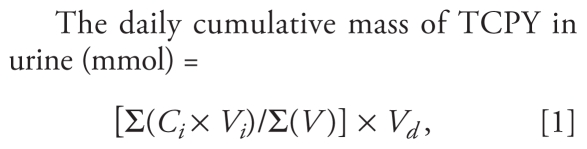


where *C_i_* = mass of TCPY measured in *i*th urine sample (millimoles per milliliter), *V_i_* = volume of urine in the *i*th urine sample (milliliter), and *V_d_* = average daily urine excretion volume (milliliter).

The cumulative TCPY excretion measured at each urine sample collection time point was calculated for each participant, and compared with the distribution of the predicted cumulative excretion of TCPY that was generated by the PBPK simulation.

### Model sensitivity analysis

A parameter sensitivity analysis was performed to identify important parameters associated with urinary TCPY excretion and tissue-specific CPF dose metrics that were important to model behavior. A local analysis was applied, where the


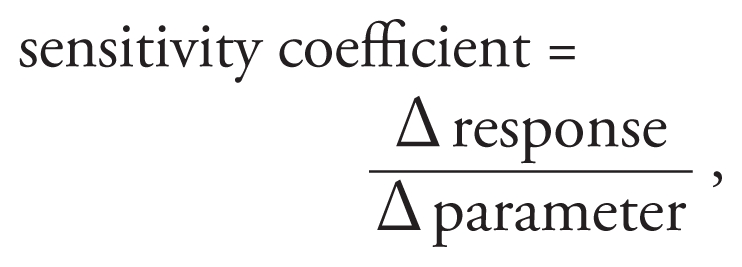


and the magnitude of the parameter change was 1% of the baseline value (Nestorov 1999; Simmons et al. 2002). Responses of interest included peak concentrations of CPF in blood and TCPY urinary excretion rates at 8 and 24 hr after an oral CPF dose of 0.5 mg/kg. All model parameters were perturbed to investigate effects on model behavior.

## Results

### PBPK models for CPF

**Figure 2:**
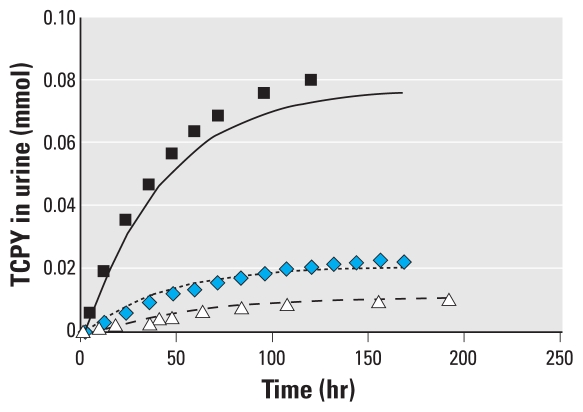
Pharmacokinetic curves for TCPY (mmol) in urine following an oral dose of 0.5 mg/kg and dermal application of 5 mg/kg in humans. The lines are the PBPK model simulations compared with the oral data from Nolan et al. 1984 (square) and Timchalk et al. 2002a (diamond) and to the dermal data from Nolan et al. 1984 (triangle).

The adult human PBPK model simulations are compared with the available data in Figure 2. The PBPK-based estimate of TCPY excretion was visually consistent with the published urinary data. This is critical for objectively interpreting urinary TCPY levels using the PBPK model. The difference in urinary TCPY levels between these two sets of human data for oral exposure is likely due to different pesticide formulations used in the respective studies. Specifically, Nolan et al. (1984) administered CPF in a sugar cube after a light meal, whereas Timchalk et al. (2002) administered CPF in a tablet. The underlying PBPK model descriptions are the same except for behavior differences in the GI tract (Table 1). When only the GI parameters were varied across studies, the PBPK model results were consistent with CPF measured in blood and TCPY measured in urine. The results of the sensitivity analysis show important parameters that may impact the PBPK model simulation and outputs [see Supplemental Material, Table 1 (doi:10.1289/ehp.0901144.S1)] and suggest that simplification of the full PBPK model to only evaluate urinary TCPY excretion is feasible. These parameters reflect areas where it is important to know the differences between adults and children depending on the questions of interest. The large sensitivity coefficients related to the tissue metrics generally correspond to metabolism and parameters associated with transport to active sites. In contrast, the TCPY urine excretion profile is strongly dependent only on the urine excretion parameters.

### PBPK model predicted and measured cumulative urinary TCPY excretion

**Figure 3:**
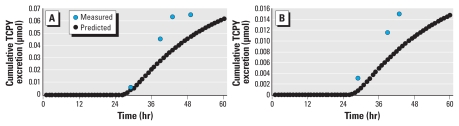
The cumulative excretion difference of TCPY between measured urinary biomarker data and PBPK-predicted values for two study participants. (*A*) Participant S1-2 (Table 3) whose PBPK input included inhalation (indoor air) and single bolus ingestion (food intake). (*B*) Participant S6-1 (Table 3) whose PBPK input included inhalation (indoor air) and single bolus ingestion (food intake).

In the 1998 study, the measured 24-hr cumulative urinary TCPY excretion among the 13 children ranged from 0 to 0.03 μmol (summer) and from 0.0025 to 0.063 μmol (fall) (Table 3). Excretions predicted by the PBPK model were consistently lower than the measured levels. However, the magnitude of underprediction, which was calculated as the predicted/observed TCPY excretion ratios, varied. In cases where CPF was only measured in a single matrix, such as in the 24-hr indoor air samples, the PBPK model simulations grossly underpredicted cumulative TCPY excretion by as many as 7 orders of magnitude (participants S3-1, S4-1, S5-2, and S7-1). This discrepancy narrowed to 2–3 orders of magnitude when CPF was detected in more than one sample matrix. In only two participants (S1-2 and S6-1) out of 26 cases in which CPF residues were also measured in the 24-hr duplicate food samples, the PBPK model predicted TCPY excretions approximate to the measured levels by 75% (Figures 3). We previously measured 350 and 12 ng/g of CPF in two separate 24-hr duplicate food samples containing cherry tomatoes (S1-2) and a composite sample of apple and carrots (S6-1), respectively (Fenske et al. 2002b).

### Absorbed dose estimates and comparisons with CPF benchmark dose

Overall, the PBPK model-predicted absorbed CPF dose was low among the study children. The two highest predicted doses (2.3 and 0.44 μg/kg/day) were associated with the two 24-hr duplicate food samplings that contained 350 and 12 ng/g CPF, respectively. For comparison, we used the measured 24-hr cumulative TCPY excretion (mmol/day), multiplying the molecular weight of CPF (350.6 ng/mol) and then dividing by the BW for each child (Table 2) to calculate a daily absorbed dose, assuming 1 mol CPF is metabolized to 1 mol TCPY that is then excreted unchanged in urine. In the absence of the PBPK models, this steady-state mass-balance approach is commonly used in converting urinary biomarker measurements to an absorbed dose. The magnitude of the discrepancy between the measured and the PBPK model-predicted urinary TCPY excretion levels carried over to the absorbed CPF dose estimation (Table 4). The calculated absorbed doses were generally higher than were their corresponding model- predicted values except for the estimates for the two children (S1-2 and S6-1) who consumed food items containing CPF residues. Urban and suburban children had marginally higher daily CPF absorbed doses than did those living in the agricultural community (one-way ANOVA, *p* = 0.07).

None of the predicted or calculated daily dose estimates exceeded the oral RfD of 3 μg/kg/day, which is a toxicologic benchmark dose established by the U.S. EPA (2002a). However, when we took into consideration a safety factor of 10, as mandated by the FQPA for the purpose of protecting children from overexposure to pesticides, the child-specific RfD of 0.3 μg/kg/day would protect children at the 90th percentile based on the model-predicted absorbed CPF doses and only at the 58th percentile level (11 of the 26 cases) using the steady-state mass-balance calculation approach.

## Discussion

The mechanistic nature of the PBPK model, including the process of linking exposure measurements of a toxic chemical to urinary biomarker data and ultimately to the absorbed dose estimation, makes its application favorable in risk assessment. It is important, therefore, to realistically characterize the targeted activities and sources that would significantly contribute to exposure, which can subsequently be used as the PBPK model inputs. Incorrect assumptions, for example, that an individual’s absorbed dose entirely results from continuous sources and steady-state levels, could attenuate the accuracy of model outputs.

In evaluating its use, it is apparent that the PBPK model has failed to predict TCPY excretion compared with the measured levels except for two children with dietary ingestion residues of CPF. This failure exposes the limitations of employing PBPK models for risk assessment in the absence of prior knowledge or high quality exposure data for model inputs. If physically measured urinary TCPY excretion in children is considered the gold standard measure of true exposure to CPF, the magnitude of underprediction by the PBPK model would be alarming. Several sources of errors could be attributed to the underperformance of the model.

First, it is possible that the PBPK model that was developed under the ERDEM platform is inadequate to simulate the ADME of CPF in children. However, we believe that this is not likely because our child-specific PBPK model was developed with parameters derived from *in vitro* systems and was validated against two different human adult data sets (Figure 2). We believe that the difference of simulation outcomes between the two data sets was due to a difference in pesticide formulations, which is irrelevant to the present study. The model sensitivity analysis showed that it was not the PBPK model but the urinary excretion rate that had the greatest impact on the dynamics of TCPY in urine. In addition, a similar PBPK/ERDEM model has been successfully applied to simulate one of the cholinesterase-inhibiting carbamate insecticides (Zhang et al. 2007).

Second, the model underprediction highlights the ongoing discussion concerning the existence of preformed OP urinary metabolites in the environment and food that may confound the results of OP pesticide exposure and risk assessment (Morgan et al. 2005; Zhang et al. 2008). The preformed TCPY in the environment and in foods may have artificially elevated the overall CPF exposure. However, the magnitude of model underprediction could not be accounted for exclusively by this cause. We previously found that approximately 36% of the fortified mass of CPF was degraded in apple and orange juices (Lu et al. 2005). A similar magnitude of incomplete degradation of OPs in fresh fruits and vegetables was also documented in a recent article, which reported that the average molar-to-molar ratio between the OP pesticides and their metabolites was approximately 2:1 (Becker et al. 2006). If the amount of preformed TCPY had been taken into account before our PBPK simulations, the predicted urinary TCPY excretion would have been very close to the measured levels for the two children who were exposed to CPF via dietary intakes.

The last possible source of error, and the likely cause of the model underprediction of urinary TCPY excretion, is the quality of the exposure data used as model inputs. The aggregate exposure assessment targeted at chemicals with short biologic half-lives, such as CPF, is prone to significant spatial and temporal variations that would result in inaccurate outcome measurements. Two observations from this study would support this conclusion. First, as previously discussed, by including the amount of preformed TCPY in the PBPK model simulation; the model-predicted TCPY excretion was still not within a reasonable range of accuracy to the measured TCPY excretion. Second, the estimates underpredicted by the model were overwhelming, even though their corresponding measured TCPY levels in children, whose excretions were underestimated, were similar to the measured levels for the two children with CPF residues in their 24-hr duplicate food samples that were close to the model predictions (Table 3). Apparently, CPF exposures in this group of children were not captured by the aggregate exposure assessment. This raises a serious question concerning the validity of applying aggregate exposure measurements as inputs for PBPK model simulation and subsequent risk assessment. Because the exposure data were collected from a study that was specifically designed to validate the PBPK model simulation, we would expect this problem to be even more prevalent and dramatic when less-structured environmental exposure data are used as the model inputs.

The PBPK model simulations have many useful features, but absorbed dose estimation is usually the ultimate application in risk assessment. Unfortunately, our results have demonstrated a potential pitfall of using a PBPK model as a tool for this purpose. Minimizing the variability associated with aggregate exposure assessment of OPs is immense and difficult to accomplish. Nevertheless, the recent regulatory change of prohibiting residential use of CPF and other OPs (U.S. EPA 1998) may have alleviated the problem by eliminating the possibility of environmental exposures to OPs, except from the dietary ingestion route. The annual report from the Pesticide Data Program, administered by the U.S. Department of Agriculture (USDA 2008) has confirmed the detection of OPs in common food commodities yearly, and recent studies have demonstrated that children are exposed to OP pesticides predominantly through dietary intakes (Lu et al. 2006, 2008). The unique exposure pathway of OPs in food and the ability of the PBPK model to simulate dietary ingestion, as demonstrated in this study, suggest that the PBPK model is still a valuable tool when the model input data reflect the best estimates of dietary OP exposures. Future improvement in assessing dietary pesticide exposure and the confirmation of the degree of spontaneous OP pesticide degradation in foods should greatly enhance the utility of PBPK modeling for pesticide-dietary risk assessment.

## Conclusions

We developed a child-specific PBPK model for CPF using models previously developed for rats and adult humans. The model was evaluated for its ability to predict urinary TCPY excretion using environmental, food, and urine data that were obtained from 13 children in a previous study. We found that the model underpredicted most of the cases except for two in which CPF residue was detected in the 24-hr duplicate food samples. Among the stipulated causes for the model’s poor performance, we believe that the potential measurement errors associated with aggregate exposure measurements are the most likely to affect the applicability of a PBPK model for interpreting urinary TCPY excretion and absorbed CPF dose estimates. The failure to predict TCPY excretion exposes the vulnerability of employing PBPK models for risk assessment without prior knowledge or assurance of the quality of exposure data used for model inputs. Regardless, we concluded that the shift from OP exposure through multipathways to dietary ingestion exclusively has made the PBPK model a valuable tool for converting dietary pesticide exposure to estimate absorbed dose estimates when model input data represent the best estimates of dietary-pesticide exposures.

## Figures and Tables

**Table 1:** PBPK model parameters that describe physiologic TCPY in rats and adult humans.

	Rat	Adult human
Partition coefficients for TCPY		
Brain:blood	23.027	15.89
Fat:blood	124.46	91.562
Kidney:blood	8.809	6.05
Liver:blood	9.648	9.95
Rapidly perfused:blood	7.452	3.94
Slowly perfused:blood	5.445	7.47

Partition coefficients for TCPY-g		
Brain:blood	1.439	1.54
Fat:blood	6.14	0.221
Kidney:blood	1.103	1.16
Liver:blood	1.046	1.27
Rapidly perfused:blood	1.071	1.05
Slowly perfused:blood	1	1.13

Glucuronidation rate		
*V*_max_3,4 (mmol/hr/kg0.75)	10	10
*K*_mm_3,4 (mmol/L)	1	1

Saturable elimination		
*V*_max_URN4 (mmol/hr/kg0.75)	10	0.74
KURN4 (mmol/L)	10	10

Parameters scaled by BW		
Liver *V*_max_ (mmol/hr/kg^0.75^)		
CPF to CPF-ox	0.08	0.08
CPF to TCPY	0.273	0.273
CPF-ox to TCPY	74.4	74.4
Blood *V*_max_ (mmol/hr/kg^0.75^)		
CPF-ox to TCPY	57	57

GI absorption rate constant (per hr)		
Stomach to portal blood	0.01	0.01^a^ (0.01)^b^
Stomach to intestine	0.5	0.5^a^ (0.5)^b^
Intestine to portal blood	0.5	0.1^a^ (0.4)^b^
Intestine to feces	0	0.4^a^ (0.1)^b^

a Values from Timchalk et al. (2002).

b Values from Nolan et al. (1984).

**Table 2:** Physiologic parameters used in the child-specific PBPK model.

Participant ID	Age (years)^a^	BW (kg)	Normalized cardiac output (L/H)^b^	Normalized alveolar ventilation rate (L/H)^b^
R1	4	15.5	23.94	14.24
R2	4	21.4	18.8	11.18
R3	4	14.5	25.17	14.98
R4	5	16.8	26.17	15.56
R5	3	13.4	22.1	13.34
R6	5	19.6^c^	23.31	13.86
S1	4	17.3	22.04	14.3
S2	6	18.2	27.83	16.68
S3	3	15	20.31	12.25
S4	4	15.5	23.94	14.98
S5	5	16.8	26.17	15.56
S6	6	22.7	23.58	14.14
S7	3	14.5	20.83	12.57

a Age rounded up to a whole year.

b Equations in Price et al. (2003) were used to calculate rates of cardiac output and alveolar ventilation; results were divided by BW^3/4^.

c Study participant did not provide a BW; value taken from *Exposure Factors Handbook*, Table 11–3 (U.S. EPA 2002b).

**Table 3:** Exposure data collected from 13 children 2–6 years of age in 1998 and their predicted and measured 24-hr cumulative TCPY excretions (μmol/day).

Participant ID	Sampling season^a^	Toy (μg/sample)	Soil (μg/g)	Indoor air (μmol/m^3^)	House dust (μg/g)	Food (μg/day)	24-hr cumulative TCPY excretion		
							Predicted	Measured	Ratio
R1	1	NA	0.025	0.01	0.28	0	3.6 × 10^−5^	1.6 × 10^−2^	2.2 × 10^−3^
	2	0	0	0	0.28	0	3.3 × 10^−5^	2.7 × 10^−2^	1.2 × 10^−3^

R2	1	NA	0	0.001	0.28	0	3.0 × 10^−5^	6.7 × 10^−3^	4.5 × 10^−3^
	2	0	0	0.001	0.28	0	3.0 × 10^−5^	5.2 × 10^−3^	5.9 × 10^−3^

R3	1	0	0	0	0.28	0	3.3 × 10^−5^	2.5 × 10^−4^	1.4 × 10^−1^
	2	0.12	0	0.001	0.28	0	1.0 × 10^−4^	2.0 × 10^−2^	5.0 × 10^−3^

R4	1	NA	0	0.003	0.28	0	3.2 × 10^−5^	1.6 × 10^−2^	2.0 × 10^−3^
	2	0	0	0.003	0.28	0	3.2 × 10^−5^	1.1 × 10^−2^	2.9 × 10^−3^

R5	1	NA	0	0.001	0.28	0	3.4 × 10^−5^	1.9 × 10^−3^	1.8 × 10^−2^
	2	0.15	0	0.003	0.28	0	1.4 × 10^−4^	5.3 × 10^−3^	2.7 × 10^−2^

R6	1	NA	0.025	0.002	0.28	0	3.4 × 10^−5^	6.5 × 10^−3^	5.2 × 10^−3^
	2	0.15	0	0.003	0.28	0	4.9 × 10^−5^	2.5 × 10^−3^	2.0 × 10^−2^

S1	1	NA	0	0.002	0.28	0	3.2 × 10^−5^	0	N.A
	2	0	0	0.002	0	39.83	4.7 × 10^−2^	6.3 × 10^−2^	0.74

S2	1	NA	0.025	0.001	0	0	2.8 × 10^−6^	5.8 × 10^−3^	4.9 × 10^−4^
	2	0	0	0.001	0.28	0	3.2 × 10^−5^	1.7 × 10^−2^	1.9 × 10^−3^

S3	1	NA	0	0.004	0	0	9.4 × 10^−8^	3.0 × 10^−2^	3.1 × 10^−6^
	2	0	0	0.002	0.28	0	3.3 × 10^−5^	3.7 × 10^−2^	9.0 × 10^−4^

S4	1	NA	0	0.001	0	0	2.4 × 10^−8^	2.8 × 10^−2^	8.5 × 10^−7^
	2	0	0	0	0	0	0	1.2 × 10^−2^	0

S5	1	NA	0.025	0.005	0.28	0	3.5 × 10^−5^	5.2 × 10^−3^	6.8 × 10^−3^
	2	0	0	0.003	0	0	8.7 × 10^−8^	1.1 × 10^−2^	8.1 × 10^−6^

S6	1	NA	0	0.001	0	2.63	1.1 × 10^−2^	1.5 × 10^−2^	0.75
	2	0.05	0	0.001	0	0	1.7 × 10^−5^	1.8 × 10^−2^	9.3 × 10^−4^

S7	1	NA	0	0.001	0	0	1.9 × 10^−8^	1.2 × 10^−2^	1.7 × 10^−6^
	2	0.05	0	0.001	0.28	0	5.2 × 10^−5^	2.2 × 10^−2^	2.4 × 10^−3^

NA, not applicable.

a Sampling season: 1, summer; 2, fall.

**Table 4:** Descriptive statistics for measured, adjusted measured, and predicted daily dose (μg/kg/day) of CPF for 13 children 2–5 years of age, summer and fall,1998.

	Daily dose, summer 1998		Daily dose, fall 1998	
Participant ID	Calculated^a^	Predicted^b^	Calculated^a^	Predicted^b^
R1	0.37	0.004	0.58	0.004
R2	0.11	0.003	0.08	0.003
R3	0.01	0.004	0.49	0.012
R4	0.33	0.003	0.23	0.003
R5	0.05	0.004	0.14	0.018
R6	0.12	0.003	0.04	0.005
S1	0	0.003	1.32	2.302
S2	0.11	< 0.001	0.33	0.003
S3	0.7	< 0.001	0.86	0.004
S4	0.64	< 0.001	0.38	< 0.001
S5	0.11	0.004	0.22	< 0.001
S6	0.23	0.440	0.28	0.001
S7	0.28	< 0.001	0.54	0.006
Descriptive statistics				
Mean	0.24	0.04	0.42	0.18
SD	0.23	0.12	0.35	0.66
Percentile				
5	< 0.0001	< 0.0001	0.04	< 0.0001
25	0.08	0.0001	0.18	0.0001
50	0.12	0.003	0.33	0.004
75	0.35	0.004	0.56	0.009
90	0.68	0.27	1.14	1.39

a Calculated daily dose using the mass balance equations and the measured 24-hr cumulative urinary TCPY excretion.

b Output from the PBPK/ERDEM simulation.
